# Autonomous indoor wayfinding for individuals with cognitive impairments

**DOI:** 10.1186/1743-0003-7-45

**Published:** 2010-09-14

**Authors:** Yao-Jen Chang, Shu-Ming Peng, Tsen-Yung Wang, Shu-Fang Chen, Yan-Ru Chen, Hung-Chi Chen

**Affiliations:** 1 Department of Electronic Engineering, Chung Yuan Christian University, Taiwan; 2 Institute of Health and Welfare Policy, National Yang Ming University, Taiwan; 3 Center for Occupational Therapy, Taipei Municipal Hospital, Taiwan

## Abstract

**Background:**

A challenge to individuals with cognitive impairments in wayfinding is how to remain oriented, recall routines, and travel in unfamiliar areas in a way relying on limited cognitive capacity. While people without disabilities often use maps or written directions as navigation tools or for remaining oriented, this cognitively-impaired population is very sensitive to issues of abstraction (e.g. icons on maps or signage) and presents the designer with a challenge to tailor navigation information specific to each user and context.

**Methods:**

This paper describes an approach to providing distributed cognition support of travel guidance for persons with cognitive disabilities. A solution is proposed based on passive near-field RFID tags and scanning PDAs. A prototype is built and tested in field experiments with real subjects. The unique strength of the system is the ability to provide unique-to-the-user prompts that are triggered by context. The key to the approach is to spread the context awareness across the system, with the context being flagged by the RFID tags and the appropriate response being evoked by displaying the appropriate path guidance images indexed by the intersection of specific end-user and context ID embedded in RFID tags.

**Results:**

We found that passive RFIDs generally served as good context for triggering navigation prompts, although individual differences in effectiveness varied. The results of controlled experiments provided more evidence with regard to applicabilities of the proposed autonomous indoor wayfinding method.

**Conclusions:**

Our findings suggest that the ability to adapt indoor wayfinding devices for appropriate timing of directions and standing orientation will be particularly important.

## 1. Introduction

Cognitive impairments range from ones that are present at birth (such as Down's syndrome and intellectual and developmental disabilities, IDD), to ones that are acquired due to some form of traumatic brain injury or illness (such as aphasia, a speech and language disorder, or amnesia), to ones that emerge through the normal aging process (such as Alzheimer's disease), to ones that arise due to complicated causes such as schizophrenia. In the US alone, an estimated 4.32 million people have intellectual and developmental disabilities [1]. Approximately 4.5 million individuals had Alzheimer's disease in 2006; this number is projected to grow to 14 millions by 2050. Aphasia impacts approximately 1.1 million individuals in North America [2]. In Taiwan, one million out of twenty-three millions of population are registered as disabled with thirty percent of them found cognitively impaired. Mentally/cognitively disabled individuals are still independently mobile, unless they are also mobility impaired. Thus there are significant numbers who could benefit from assistive technology for wayfinding.

While people without disabilities often use maps or written directions as navigation tools or for remaining oriented, this cognitively-impaired population is very sensitive to issues of abstraction (e.g. icons on maps or signage) and presents the designer with a challenge to tailor navigation information specific to each user and context. For example, some dementia patients may suffer from spatial disorientation at unfamiliar places or forgetting intended destinations [3]; people with traumatic brain injury (TBI) or intellectual and developmental disabilities may not be able to recall clues of the routes they once firmly trained to acquire [4,5]. Current methods in social services for aiding people with wayfinding are labor-intensive [6]. For example, job coaches at several Taipei-based rehabilitation institutes, who work with individuals with mental impairments to support them in learning new jobs and maintaining paid employment, may work for weeks helping a person learn how to travel to and from work. Even then, the individual may at times still require assistance of one form or another. While en route to the work, the person needs to be reminded by phones from the supporting group, or followed by the job coach invisible to the person, in order to keep things safe and in control.

With the capacity to move and the desire to be socially included, the system developed in this study is targeted on those mentally/cognitively disabled individuals who are independently mobile but have difficulties reaching the expected destination. This paper describes an approach to providing distributed cognition support of indoor navigation for persons with cognitive disabilities. A prototype was built and tested in field experiments with real subjects. RFID tags were placed at decision points such as hallway intersections, exits, elevators, and entrances to stairways.

The contributions of the paper include the following: (1) both exploratory and comparative study of an indoor wayfinding system for participants with cognitive impairments; (2) use of RFID technology to support wayfinding without a shadow team; and (3) adaptation of a simplified task load index (TLX) for subjective assessment of user experiences. The paper is organized as follows. In the next section, we survey the state of the art in the wayfinding research for individuals with cognitive impairments. Then, prototype design is presented. Implementations and results are shown with follow-up discussions. The paper concludes with some final remarks.

## 2. Related Work

The growing recognition that assistive technology can be developed for cognitive as well as physical impairments has led several research groups to prototype wayfinding systems. A resource-adaptive mobile navigation system [7,8] was studied for both indoor and outdoor environments, although it was not specially design for people with disabilities. Cognitive models were built to study human wayfinding behaviors in unfamiliar buildings and salient features of route directions were identified for outdoor pedestrians [9,10]. Kray [11] proposed situational context for navigational assistance.

Baus et al. [12] developed auditory perceptible landmarks for visually impaired people and the elderly people in pedestrian navigation and conducted a field experiment on a university campus. Goodman, Brewster, and Gray [13] showed that an electronic pedestrian photo-based navigation aide based around landmarks was more effective for older people than an analogous paper version. Opportunity Knocks (OK) [14] and other similar work form the University of Washington [15] provided text-based routing directions for users with GPS-enabled cellular phones. It can issue user errors if there is deviation being detected. The Opportunity Knocks experiment was based on one single outdoor user. Furthermore, Opportunity Knocks used a hierarchical Dynamic Bayesian Network model in the inference engine to continuously extract important positions from GPS data streams in outdoor navigation.

Sohlberg, Fickas, Hung, and Fortier [5] at the University of Oregon compared four prompts modes for route finding for cognitively impaired community travelers. It was found auditory modality was better than text or image modality in outdoor use of PDAs because image and text on the PDA screen is difficult to read under the sun, especially for subjects with poor vision in their field study. A "Wizard of Oz" approach instead of a context-aware implementation was used for sending navigation information. Researchers at the University of Colorado have implemented a system for delivering just-in-time transit directions to a PDA carried by bus users, using GPS and wireless technology installed on the buses [16].

The Assisted Cognition Project at the University of Washington has developed artificial intelligence models that learn a user behavior to assist the user who needs help [15]. The system was tested with success in a metropolitan area. Later a feasibility study [17] of user interface was conducted by the same team, who found photos are a preferred media type for giving directions to cognitively impaired persons who navigated indoors, in comparison with speech and text. They also used a "Wizard of Oz" approach to decide when to send photos from the shadow team.

## 3. A Rfid-Based Wayfinding Design

**Figure 1:**
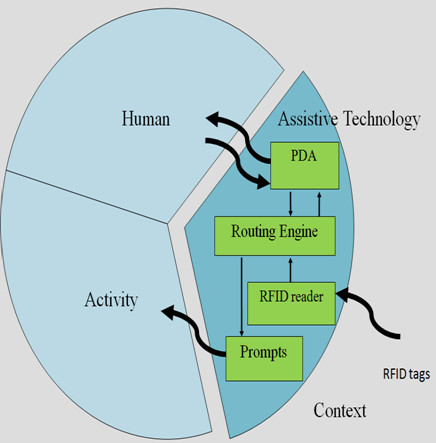
**Model of wayfinding devices**.

As informed by the human activity assistive technology (HAAT) model [18], an assistive solution has four components: the human, the activity, the assistive technology, and the context in which the first three integrated factors exist. In light of the HAAT model, our prototype called U-Service DOG (Ubiquitous Service for Direction Guide) is designed to assist with navigation for individuals with cognitive disabilities. It consists of PDA user interfaces, RFID tags and readers, and a routing engine. See Figure 1. Each component will be described in the following.

### User Interface Design

People's spatial abilities depend mainly on the following four interactive resources: perceptual capabilities, fundamental information-processing capabilities, previously acquired knowledge, and motor capabilities [19]. These abilities are a necessary prerequisite for people to find a way from an origin to a destination. However, for people with severe cognitive impairments, the first three resources are generally limited. Therefore, the proposed system provides multimedia cues for them to use as environmental information, and a PDA for them to process representations of spatial knowledge in order to move through the environment. In addition, Passini [20] studied the communication aspect of wayfinding design. In terms of wayfinding communication, designers have to respond to three major questions: what information should be presented, where and in what form. Passini further pointed out that a key rule of environmental perception is that information is not seen because it is there but because it is needed. During wayfinding, people will select that information which is relevant to their task. An analysis of decisions made by subjects who tried to find a destination, showed that they tended to perceive information when it was directly relevant to the behaviors associated with an immediate task and did not perceive information irrelevant to the immediate task even if it might be useful later on. Therefore, spatial abilities are sensitive to perceptual information, and in particular the time and place to receive it.

The design draws upon the requirements based on interviews with nurses and job coaches at rehabilitation hospitals and institutes. Previous work of Passini with dementia [20] has shown that patients with dementia show marked cognitive wayfinding deficiencies. They tend to have significantly reduced cognitive mapping abilities. They are not able to make wayfinding decisions requiring memory or inferences while they may still be able to make decisions based on explicit architectural information and directional signs. They can no longer develop decision plans, and can only operate from one decision point to the next so that they can be mobile and as autonomous as possible. This motivated us to use a prompting device to provide directional guidance at decision points. In light of Passini's findings, the proposed wayfinding system uses the PDA to provide the signage on the screen in the format of pictures or videos when individuals with cognitive impairments approach decision points.

### RFID for Distributed Cognitive Aid

To provide indoor navigation assistance, the user position needs to be determined first. Wi-Fi, Bluetooth, 2G, 3G, ultrasonic waves, and lasers are among the competing choices for indoor positioning with which the major issue is the trade-off between accuracy and cost [21]. Except very expensive equipment such as lasers and ultrasonic waves, the state of the art technology [22] with 3 meters in errors makes it difficult to apply in indoor positioning because the user may have gone into an incorrect direction until he receives any navigation assistance. In our study, radio-frequency identification (RFID) tags [23] are used for the purpose of wayfinding. RFID is an automatic identification method, relying on storing and remotely retrieving data using devices called RFID tags or transponders.

There are two kinds of RFID tags. Active tags can operate remotely within one to two meters without visual contact. However, they are expensive and battery operated. On the other hand, passive tags work without battery because they are radio charged momentarily by a reader. They can be packaged in a rugged form factor, cost less than a dollar each peace and they are maintenance free. For large buildings with thousands of nodes of deployment, the cost becomes a critical issue. Therefore, we adopt passive RFID tags to trigger navigational cues.

A RFID tag is placed at each decision point which is any physical position where the individual is presented with a navigational choice. Decision points where navigational choices must be made may be doorways, corners, or intersections of corridors. In many situations such as straight corridors in indoor environments, no RFID tags need to be placed in the middle since there are no changes of directions. Therefore, RFID tags are not densely distributed everywhere. The users need to visually indentify and locate a tag which is mostly on the wall before their PDA with an add-on or built-in RFID reader can interact with the tag in a short distance, usually less than 5 centimeters. To reduce the cognitive load of identifying tags in surrounding areas, the tag positions are not only adjusted to be more noticeable but also attached to flashing blue LEDs to further increase opportunities to get read.

### Delivery of Navigational Cues

**Figure 2:**
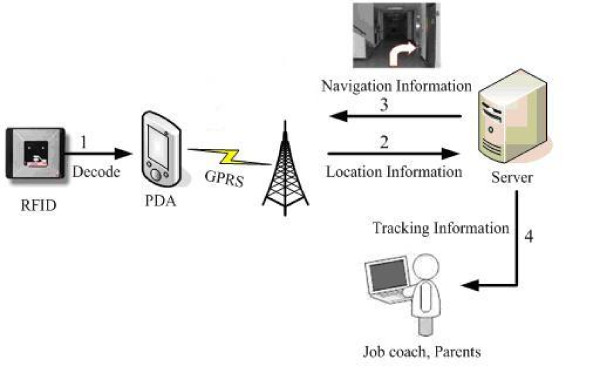
**Architecture and system interaction of the wayfinding prototype**. Architecture and system interaction of the wayfinding prototype. (1) By sensing the RFID tag, the user PDA determines the current location of the user. (2) Location information is sent over GPRS to the server. (3) The server decides which photo to send to the user.

**Figure 3:**
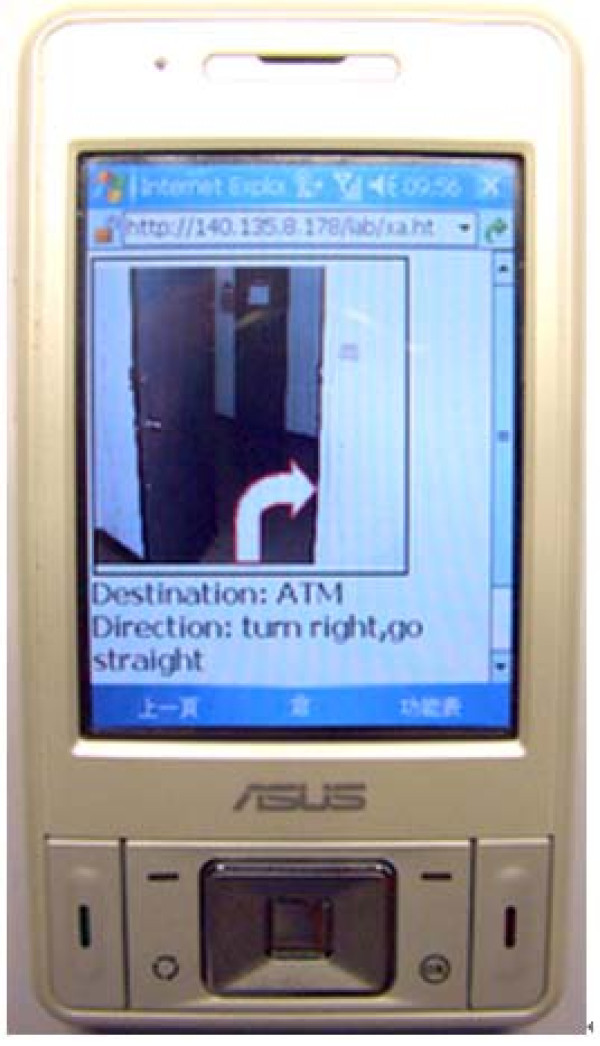
**An augmented photo**. An augmented photo overlaid with an arrow showing the just-in-time direction on the wayfinding PDA.

The proposed PDA shows the just-in-time directions by displaying photos, thus eliminating the need of a shadow team behind the user and reducing the burden on care-providers. The architecture of the proposed system is shown in Figure 2. Although in the laboratory we tested connectivity options including GPRS, Wi-Fi, 3G, and 3.5G and measured each performance, we used only GPRS in the field experiment. We didn't preload the PDA with all the navigation pictures used in the field test to emulate the situation that individuals with cognitive impairments download the pictures on demand when visiting new indoor environments. Therefore, the PDA constantly communicates with the server despite of the cached memory of the PDA. However, for navigation photos that are cached, their download time is saved because of direct fetch from the memory of the PDA. See Figure 3 for a sample picture downloaded on the PDA screen.

### Dual Interface

**Figure 4:**
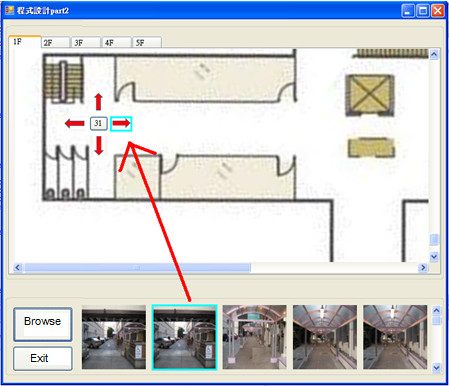
**A decision point**. A decision point labeled with node 31 is to be loaded with four photos, one for each direction. A photo facing the east is currently highlighted by clicking the selected photo in the bottom row.

Assistive devices for individuals with cognitive impairments often need a dual interface for their care providers to program the device. End user programming was confirmed as one of the key system requirements during user interviews because it can eliminate the need to involve rehabilitation engineers or technicians. For dual interfaces designed for the wayfinding system, see Figure 4.

### Routing

**Figure 5:**
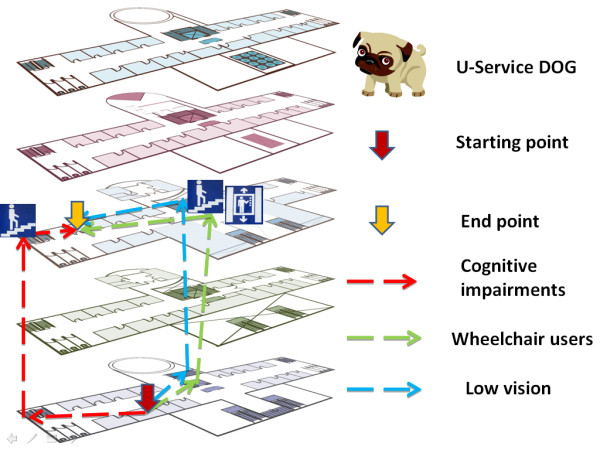
**Routing for individuals with multiple special needs**. Routing for individuals with multiple special needs: users with cognitive impairments, wheelchair users, and users with low vision

Routing is a key feature of wayfinding devices. The details of the routing algorithm were described in our earlier work [6]. See Figure 5 for three routes planned for individuals with various disabilities. In the prototype, the Dijkstra algorithm, a time-honored graph theoretic method [24] is applied for routing. Oftentimes, a user can deviate from the correct route because he misses a tag, misinterprets the pictorial prompt, or simply gets distracted. Again the system employs the Dijkstra algorithm to handle situations in which detours have been taken during the way-finding process. The system simply uses the tags on the detours to reroute a new path.

Navigating in indoor symmetric environments such as many public buildings can be especially a challenging task. It is true for people with or without cognitive impairments to get lost if there is symmetry when they stand still and look around. Fortunately, when they navigate indoors, we can provide directions for them when they face symmetry according to the node they just visited. For example, when an individual with cognitive impairments steps out of an elevator and encounters both a left corridor and a symmetric right corridor, the navigation photo shows the turn to take next before exiting the elevator. Similarly, when an individual moves along a hallway and then hits two symmetric corridors, we arrange a photo to indicate which turn to take before entering the turning point by placing an RFID tag.

When people with cognitive impairments decide to change their minds in the middle of the planned route, they simply go back to the main menu of the navigation system on the PDA and choose a new destination. We didn't provide the "abort" function per se. Even if they decide to go back to when they come from, they set the origin as the new destination.

## 4. Experimental Results

The PDA used in the experiment is an ETEN X800, equipped with a screen size 320*240, Wi-Fi 802.11 g, Bluetooth, GPRS/HSDPA and an ISO 14443A RFID reader the scanning range of which is about 10 cm. The size of photos is the range of tens of Kbytes in JPEG format. The user interface for rendering the photos is programmed on Microsoft IE Mobile for Windows Mobile 6.0. The server is an Intel-based PC for authenticating the users, planning a trip, serving photos upon requests from PDAs, and receiving timestamps for each position visited. The connectivity was provided by GPRS in our experiment, although 3G, HSDPA, and 802.11 g Wi-Fi networks were also used during the development stage. The download time of a prompting photo for various types of connectivity, each based on multiple measurements, is shown in Table 1. The delay in navigation update caused by the tag identification, GPRS connection and image rendering is mostly around 4.0 seconds.

**Table 1:** Download time of a navigation photo of size 31 Kbytes.

Type of Connectivity	GPRS	3G	3.5G, HSDPA	Wi-Fi 802.11 g
Max. Speed	114 kbps	384 kbps	1.8 Mbps	54 Mbps

Measured Speed (kbps)	62.0	88.6	104.8	548.0

Avg. Time (sec)	4.0	2.8	2.3	0.5

* Average time is based on 5 times of measurements

Photos can be stored on PDA ahead of time and invoked immediately when needed. Occasionally, the quality of photos has to be enhanced and photos are retaken. Therefore, the PDA is designed to remain connected when in use so that the most updated photos can be retrieved. Downloaded photos are locally cached for repeated use. This could potentially save communications energy and perhaps cost while reducing significant amounts of response time.

### 4.1 Settings

**Figure 6:**
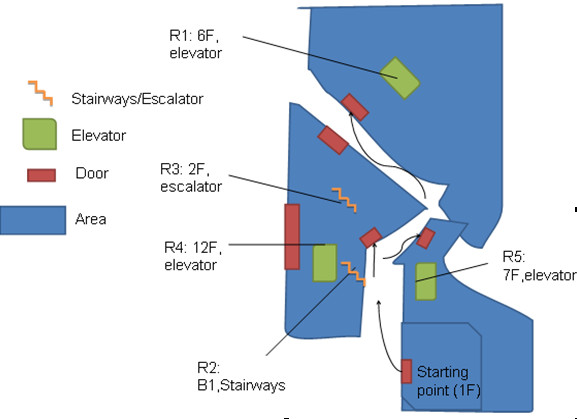
**The five routes used in the experiments**.

Five routes in different combinations of stairways, elevators, and turns were used in the study. The routes exhibited various complexities, which are summarized in Table 2. Route 1 (R1) starts from the Rehabilitation Center, which is located on the ground floor, to the Employee Library, which is at the sixth floor of the Tech Building (Figure 6) and involves using an elevator in the middle. Route 2 involves taking the stairs down one flight and Route 3 involves taking the stairs up one flight. Route 4 involves using an elevator and then 4 turns on the same floor to hit the destination.

**Table 2:** Route profiles and complexities

Route ID	Destination	Vertical movements	Turns	#RFID to stop by
R1	Library, Tech Building	1F~6F	3	7

R2	Warehouse, Central Building	1F~B1	6	6

R3	HCI Lab, Central Building	1F~2F	5	7

R4	Gym, Central Building	1F~12F	4	7

R5	Library, Administration Building	1F~7F	3	8

### 4.2 Volunteer Recruitment Protocol

Participants were recommended by the participating rehabilitation institutes and screened according to severity of cognitive impairments, the ability to remain oriented, and severity of loss in short-term memory. To prove the effectiveness, priorities were given to medium and low functioning individuals as opposed to high functioning ones. Moreover, screening also took into account the ability to operate the PDA and understand its feedback. An assessment was made to decide the qualification as subjects. Table 3 lists the basic profiles of the six participants with sensitive and irrelevant data omitted.

**Table 3:** Profiles of six participants

ID	Gender	Age	Education	Syndromes
1	M	26	High School	Intellectual and developmental disabilities (IDD)

2	F	21	High School	IDD, Epilepsy

3	M	76	College	Parkinson's Disease

4	M	37	High School	Dementia

5	F	19	High School	Schizophrenia, IDD

6	F	47	Elementary School	Schizophrenia

The user group in our study consists of individuals with differing types of cognitive syndromes, ages, and physical conditions in evaluating effectiveness and the appropriateness of the proposed system. Participant 1 has IDD and mild difficulties in memorizing routine procedures in his workplace. He occasionally gets lost and has to call for help by cellular phones. Participant 2 has also IDD and her epilepsy has significantly negative impacts on her cognitive abilities. She also has problems holding things steadily, including the PDA to be used in the experiment. She is on a paid job working in a restaurant kitchen where travel in large areas is not required. Participant 3 has the Parkinson's disease in addition to depression. He is always accompanied by his family no matter where he travels. Participant 4 has dementia and is forgetful about routes or work procedures. He has not been under employment since a car accident happened to him some years ago. Getting a job some day is his wish. Participant 5 has IDD and schizophrenia, which make her unable to distinguish ambient sounds from those imagined from within. Participant 6 has schizophrenia. Her family hires a caregiver to accompany her all day long. During the experiment, she was found to become playful with the PDA in hand and enthusiastic.

### 4.3 Field Experiments

All the six subjects were first-time PDA users. Participants were shown the device and trained before the experiments. They practiced how to touch the buttons on the screen, how to orient the PDA to read the RFID tag, and when to pay attention to the photos on the screen. They also asked questions they came up with and we tried to answer and explain until they felt comfortable to start taking the routes. Such pre-test session normally took 10 to 20 minutes. Afterwards, they were led to the starting location of each route and given the task of following the device's directions to the destination. The routes were all unfamiliar to the subjects.

**Figure 7:**
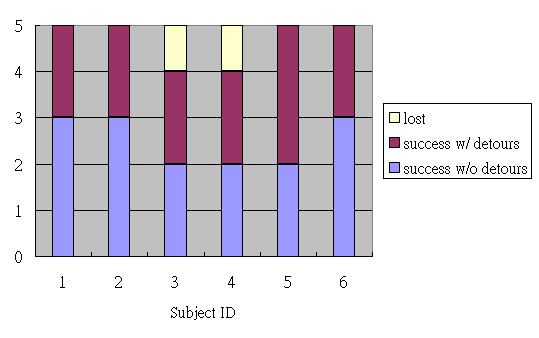
**Experiments of six cognitively impaired participants taking the five routes**.

The computational cost to determine a path given a large scale deployment is manageable because the Dijkstra algorithm can efficiently handle sparse matrices of nodes in buildings. In our experiments, computation of routes took time in the order of 0.001 seconds on the PDA for a map with 364 nodes. In Figure 7, we summarize the experimental outcomes based on the observations of the prototype design team. In the 30 trips made by 6 cognitively impaired participants taking 5 routes, there were 15 successes without detours and 13 successes with detours. The ratio of successful wayfinding was 93%. When participants took detours, they were rerouted by the wayfinding device. In the 13 successes with detours, participants had two additional reads of RFID tags for each detour, one in the detour and the other en route. Participant 3 failed to complete the route involving an elevator where he seemed to have difficulty understanding the photo that told him to press a button on the panel. In other words, he was stuck. However, on the remaining routes that also involved elevators, there was no further problem. For participant 4, he bypassed a tag without scanning it on Route 1, which resulted in a detour to an exit of the building. The PDA didn't reroute him back because of no outdoor RFID tags.

After the exploratory study, we designed control experiments to see whether the performance difference with and without technology is statistically significant. Furthermore, we designed comparative experiments to test the efficacy by observing behaviors on an alternating treatments design study.

### 4.4 Control Experiments

Control experiments were conducted by asking participants to take one route that was felt most comfortable among the five routes. Before the control experiments, participants were first asked whether they had confidence using learning transference they just acquired with PDAs to navigate without PDAs. During control experiments, a paper print-out with a set of pictures on the map of the routes was used. The control route was taken without PDAs so that we could compare the proposed system to the low-tech baseline. After the low-tech experiment was finished, a participant was asked to navigate the same route using PDA. We didn't plan a full scale control experiment by asking test subjects to go through the same process for every route because most of them had only limited physical strength.

Table 4 summarizes the control experiments. Each individual was asked whether s/he could make it without PDAs. The self-estimation is recorded in the "self-confidence" field. In control experiments, only two of the six participants succeeded compared to all the six who succeeded on the same route taken earlier with a PDA.

**Table 4:** Control Experiments.

Participant	Route picked	Self-confidence (Yes/No)	Success with print-out	Success with PDA
1	R4	N	N	Y

2	R5	Y	Y	Y

3	R5	N	N	Y

4	R5	Y	N	Y

5	R5	Y	Y	Y

6	R3	N	N	Y

Ratio (when applicable)		50%	33%	100%

Due to insufficient short-term memory, learning transference didn't help much for participants 1, 3, 4, and 6. Although participant 4 initially had self-confidence, he still didn't make it with assistive technology. The results showed the performance with assistive technology was better than that with the low-tech baseline. The control experiments were concluded by repeating the chosen route with the PDA for navigation. The results were consistent between the first time use of PDAs and the second. Therefore, the control failures with participants 1, 3, 4, and 6 were not attributed to physical fatigue.

### 4.5 Task Load Measurement

Besides technical evaluation, subjective workload measurement is also important to the success of the system and adoption of the assistive device. To evaluate the task load subjects may have experienced during device use, we adopt Hart and Staveland's NASA Task Load Index (TLX) method [25]. NASA TLX includes 6 indices: mental demand, physical demand, temporal demand, performance, effort, and frustration. Considering the reading and verbal limitations with our subjects, TLX-based assessment was conducted in the form of oral interview. In the meantime, 21 gradations have been simplified and reduced to only 5, i.e. 1 to 5, representing very low, somewhat low, neutral, somewhat high, and very high. The survey results are summarized in Table 5.

**Table 5:** Subjective assessment of task load on PDA users

TLX Index	Test subjects (ID)					
	
	1	2	3	4	5	6
Mental demand	1	1	1	3	2	2

Physical demand	1	1	2	3	2	2

Temporal demand	2	2	1	2	2	2

Efforts	1	2	2	3	2	2

Frustration	2	1	2	2	2	2

Performance	5	5	5	4	4	4

In this study, the subjects unanimously found mental, physical demands and efforts to operate the device low or very low except that participant 4 considered them neutral. In addition, no individual felt rushed to meet the expected level of performance. The pace of the task was not hurried either. The performance of the proposed system was considered high or very high. During the interviews, all the participants felt comfortable recommending the system to their friends. No significant frustration was experienced by the participating users.

### 4.6 Comparative Study

After the exploratory and control studies were concluded, we invited our test subjects for a comparative study to validate robustness of the proposed method against other methods. The study design combined a multiple-probe across subjects design [26] with an alternating treatments design [27]. The multiple-probe design allowed us to demonstrate a functional relation between introduction of picture-based prompts and increases in the percentage of wayfinding tasks completed correctly. The alternating treatments design allowed us to compare the relative effectiveness of shadow team experiments and autonomous wayfinding.

Participant 1 and participant 4 agreed to take part in a three-week experiment. During the first week, a baseline measurement was accomplished with a subject carrying the map of routes one at a time before walking to the destination. For each session, all the five routes were tested and statistical results in terms of success rates were collected. During the second week, a shadow team approach [5,17] was taken. Subjects carried a PDA which received navigation cues from the PDA of a shadow team whenever a decision point was approached. In other words, the timing of prompts was controlled by the shadow team instead of the context in the environments. In the last week, our proposed method of autonomous wayfinding was used and subjects carried the device for indoor navigation.

**Figure 8:**
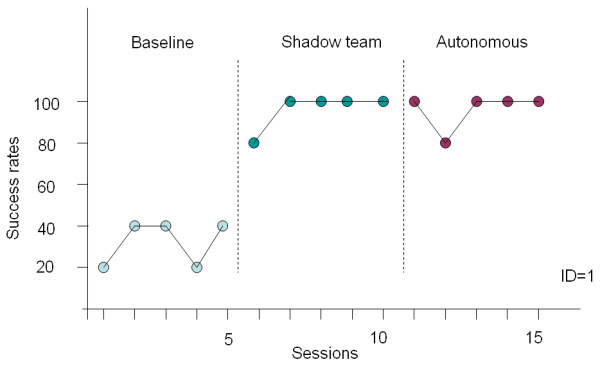
**The study of participant 1 in three phases**.

**Figure 9:**
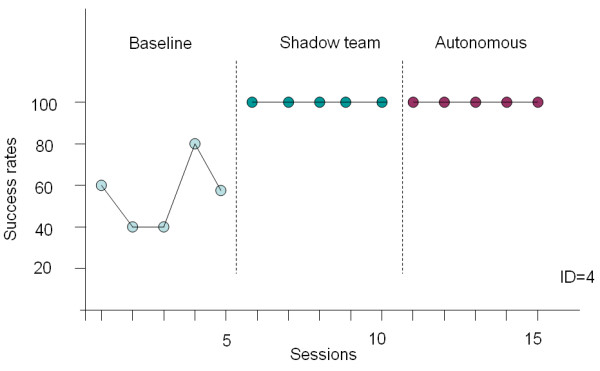
**The study of participant 4 in three phases**.

The experiments resulted in a total of 30 sessions in 3 different navigation strategies with 15 sessions for a subject. The statistics is depicted in Figure 8 and Figure 9. For the two subjects, the baseline achievements varied from 20% to 40% for participant 1 and from 40% to 80% for participant 4. The performance difference between the baseline and the shadow team is statistically significant (ID 1: p = 0.0001, t = 10.1193, df = 8; ID 4: p = 0.0004, t = 5.8797, df = 8). The performance of the shadow team approach in terms of success rates improved significantly for the two subjects. With the wayfinding system, the performance difference between the PDA and the baseline is statistically significant (ID 1: p = 0.0001, t = 10.1193, df = 8; ID 4: p = 0.0004, t = 5.8797, df = 8). The high success rates validate the effectiveness of the navigation cues and interface design of the PDA. The results in the autonomous wayfinding strategy indicate that the performance is as good as the shadow team approach and that the navigation cues can be triggered by users themselves sensing RFID tags without a shadow team behind. Participant 4 was able to achieve independence in indoor wayfinding using the proposed prompting system for five consecutive sessions at 100% success rates while participant 1 had four sessions with 100% success and an occurrence of 80%.

### 4.7 Discussions and Implications

Participants' self-estimation is found to be consistent with the experimental outcome, except participant 4 who thought he could make it. There were two participants, namely participants 3 and 4, who ran into difficulty and deviated from the correct path, respectively. Participant 3 had difficulty understanding how to interpret a photo to take an elevator to a desired floor. We modified the photo he didn't understand so that it showed a person's hand pressing the desired button instead of just the button alone. In the case experienced by participant 4, we learned that some turns seemed to be easier to be neglected. For those turns, we adjusted the tag positions to be more noticeable.

We further discuss the impact of carryover bias in the study. If the effect of a treatment continues after the treatment is withdrawn then the response to a second treatment may well be due in part to the previous treatment. This, so called, carryover effect may bias any type of study in which subjects are tested more than once. One way of reducing the amount of the carryover bias should require a sufficient washout period between two tests on the same subject. However, due to physical strength and personal safety concerns, we are allowed only an afternoon of time for the two tests. Therefore, the carryover bias may indeed remain unpreventable. Our strategy is to favor the efficacy of control experiments by first conducting the technology assisted experiment and then the control experiment for each subject. Due to carryover effects of learning transference, what we obtain in the control experiments are actually upper bounds of their efficacy. By doing so, we make it harder for the proposed assisted technology to outperform the baseline.

In the control experiment, the subject was allowed to choose their preferred route. The rationale behind it is similar. Due to limited time and physical strength, we didn't ask subjects in the control experiment to repeat every route they navigated with assistance of technology. We let them choose their preferred route instead. The performance of the control experiment should therefore be interpreted as an upper bound.

A service provider who eventually deploys a system like this has to ensure that a tag remains functional over time with proper packaging and installation so that there isn't much a chance that it could fall off the wall, get covered by something, or become difficult to notice in some way. Furthermore, a primary security concern surrounding RFID technology is the illicit tracking of RFID tags. Tags which are world-readable pose a risk to personal location privacy. In our study, RFID tags are not carried by the individuals but fixed like road signs. Therefore, there are no security issues on the tags.

Social stigma was once considered a potential issue before the experiment was started. Interacting with special signage at decision points was supposed to single out some potential users of the system and reveal the condition of the user to all bystanders. However, our field experiments in a crowded building complex revealed that it was not a real issue. Bystanders were rarely attracted to watch our subjects using their wayfinding PDAs, except that only two seemed curious about the signage deployed on the routes and mere one of them bothered to ask what it was. PDAs have been around for several years and people tend to see them like ordinary cellular phones. Therefore, using PDAs as wayfinding devices did not become a target of social stigma. We observed low initial reservations and resistance users exhibited about the device and the technology.

### 4.8 Limitations and Future Directions

One limitation to this study was the small number of participants. Originally, 8 participants were recruited. However, one participant became ill and missed several weeks of occupational training; the other left his job coach because of decreasing willingness to become employed. Therefore, replication studies are needed to confirm the findings provided here when used with increasing numbers of users with moderate and severe cognitive disabilities.

There are some limitations to the PDAs and user interfaces. PDAs are fragile and not weather proof. Therefore, protective measures need to be taken to keep them in good maintenance from frequent use. Strong sunshine can make the screen hardly viewable. Fortunately, it is not a problem in indoor wayfinding. Form factors are also an issue. Although small and thin PDAs are easier to carry with, small screens are less useful for photo rendering. The use of RFID readers with a short range implies the user standing close to a tag. Future research directions include the alternative technology such as active RFID tags or Wi-Fi beacons to determine the user's actual position in the environment (e.g. [28]), which can potentially reduce the cognitive load and possibility for users of the system to miss a tag en route.

## 5. Conclusions

This paper presents a simple, effective RFID based system for indoor wayfinding. A small user study involving individuals with cognitive impairments investigated its performance in exploratory, control, and comparative experiments with the PDA equipped with autonomous routing capabilities and context sensing. We hope this study adds to the limited literature of indoor wayfinding that was based on distributed cognition without relying on a shadow team. The results can potentially reduce the burden on care-providers. Given the evidence base of empirical data, the system is an appropriate candidate to address the wayfinding needs for people with mild or more moderate forms of impairments in various types of cognitive syndromes. However, the success ratio can depend on the extent to which participants are impaired with mental disabilities, the complexity of routes, the degree of received training and self-practices, and the distractions the participants may encounter.

## Competing interests

The authors declare that they have no competing interests.

## Authors' contributions

YJC led this work, designed the experiment, and recruited care providers from hospitals. YJC also drafted the manuscript. SMP, YRC, and HCC performed the measurements of all participants, data analysis, and statistical analysis. TYW participated in the field work and coordination of the field study and assisted with drafting the manuscript. SFC assisted with recruiting participants with cognitive impairments and caring during the experiments. All authors read and approved the final manuscript.
